# Experimental Characterization and Correlation of Mayer Waves in Retinal Vessel Diameter and Arterial Blood Pressure

**DOI:** 10.3389/fphys.2018.00892

**Published:** 2018-07-13

**Authors:** Steffen Rieger, Sascha Klee, Daniel Baumgarten

**Affiliations:** ^1^Institute of Biomedical Engineering and Informatics, Technische Universität Ilmenau, Ilmenau, Germany; ^2^Institute of Electrical and Biomedical Engineering, UMIT – Private University for Health Sciences, Medical Informatics and Technology, Hall in Tirol, Austria

**Keywords:** static vessel analysis, Mayer waves, cross-correlation, retinal vessels, vessel diameter, blood pressure, vasomotion

## Abstract

Retinal vessels show various biological temporal variations that can impact diagnosis using a static vessel analysis. In this study, Mayer waves in the retinal vessel diameter and arterial blood pressure (BP) signals were characterized, and the temporal correlation between these two modalities was investigated. The arterial and venous vessel diameters and arterial BP were recorded simultaneously on human subjects. The obtained vessel diameters showed vasomotion amplitudes over time. The vessel diameter and BP signals contained multiple signals in the frequency domain and varied over time. The signal characteristics were similar within the measurements. The BP and arterial and venous vessel diameters were correlated. The highest correlation values between the signals were observed for shifts of 1 or 0 periods. The spectrum and amplitudes of the Mayer waves showed a high variability. The Mayer waves in the retinal vessel diameters showed the same characteristics as those in the arterial BP. A temporal dependency between the oscillations in the arterial BP and retinal vessel diameters was shown.

## Introduction

Static vessel analysis (SVA) is a noninvasive tool for the risk evaluation of cardiac diseases based on retinal vessel diameters analyzed in fundus images. Studies have shown a dependency between the artery-to-vein ratio (AVR) and the risk for cardiovascular diseases. Specifically, correlations to hypertension (Ikram et al., 2006a,b), stroke (Wong et al., 2001), and cardiovascular mortality (Wang et al., 2007) have been verified. Correlations to diabetes (Wong et al., 2002) and obesity (Wang et al., 2006; Hanssen et al., 2012) have also been shown.

Static vessel analysis is usually executed according to the paradigms introduced by Hubbard et al. (1999). The diameters of multiple retinal arteries and veins are measured in a specified area around the optic disk in a single fundus image. The artery diameters are combined into a central retinal artery equivalent (CRAE), and the vein diameters are combined to a central retinal vein equivalent (CRVE) according to the formulas developed by Parr and Spears (1974a,b). The AVR is calculated as the quotient of CRAE and CRVE.

Retinal vessels are biological systems, which contain many biological variations. However, SVA only uses a snapshot of this dynamic system. A previous study has shown a SD for AVR of 0.015 (at a mean AVR of 0.844) between three photographs taken during one visit. This demonstrated good reproducibility (Neubauer et al., 2008). These measurements were obtained from subjects with an age of 64 ± 9.12 years (mean ± SD) using a modified SVA measurement protocol involving only the largest vessels. Other studies have reported a decrease in activity with an increasing age (Jarisch et al., 1987). This suggested that the variability between the multiple photographs could be higher for younger subjects. Investigations on younger subjects with an age of 29–57 years (mean of 34.6 years) showed a diameter change in the artery and vein of 3.46 and 4.82%, respectively, by pulsation and 3.71 and 2.61%, respectively, by vasomotion (Chen et al., 1994). Pulsation refers to heartbeat-related variations, and vasomotion indicates slower changes in the vessel diameters. Some working groups differentiate between vasomotion and vasomotor changes according to their origin; however, in this study, vessel diameter changes were considered vasomotion. The reduction of pulse-related changes in the diameter using electrocardiograph (ECG)-synchronized fundus photography has been examined (Dumskyj et al., 1996). However, methods for reducing the influence of vasomotion have not been published.

Mayer waves are low-frequency (LF) oscillations in the cardiovascular system. In 1876, Sigmund Mayer described spontaneous oscillations in arterial blood pressure (BP) (Mayer, 1876). These oscillations can be seen in different modalities. Studies have measured Mayer waves in arterial BP (Aono et al., 1996; Kiviniemi et al., 2010), heart rate variability (HRV) (Aono et al., 1996; Camm et al., 1996), blood flow (Killip, 1962), near infrared spectroscopy (Yucel et al., 2016), and retinal arterial vessel diameters (Vilser et al., 2002; Bek et al., 2013).

Seydnejad et al. grouped these oscillations into three major components: a high frequency (HF) of approximately 0.25 Hz, a LF of approximately 0.1 Hz, and a very low frequency (VLF) of approximately 0.04 Hz. The HF oscillations were synchronous with respiration. The LF oscillations were attributed to the sympathetic activity and controlling action of cardiovascular regulation, and the VLF component is supposed to originate from the vasorhythmicity thermoregulatory system or the humoral regulations. The LF and VLF waves are usually modeled as an oscillating closed-loop regulation mechanism. (Seydnejad and Kitney, 2001)

Various frequency ranges of the VLF and LF waves have been reported. A study determining the spectral power peaks in BP showed a high variation for the frequencies (Vermeij et al., 2014). A structured analysis of Mayer waves related to the frequencies and amplitudes in retinal vessel diameters has not been published.

Vasomotion is an important source of variation in vessel diameters. For a reliable and reproducible measurement using SVA, reducing the uncertainties resulting from vasomotion is required. In this study, we evaluated the variations in the frequency ranges of Mayer waves in the arterial and venous retinal vessel diameters as well as in the arterial BP in humans. For the dynamic vessel diameter measured by a retinal vessel analyzer (RVA) and arterial BP data, the influence of the Mayer waves on the retinal vessel diameters and BP was analyzed. The frequencies and amplitudes of the Mayer waves were characterized. The temporal dependency between the retinal vessel diameters and the arterial BP was determined. This study hypothesized that the Mayer waves in these modalities contained the same characteristics and a temporal correlation. Various cardiovascular regulation mechanisms were investigated and modeled; however, the retinal vessel diameter was not considered. For a broad clinical application of the SVA, uncertainties have to be known and precision has to be increased. This study provided an important contribution to the further development of SVA.

## Materials and Methods

### Study Design

We performed measurements on 15 young and healthy human subjects (11 male, 4 female) with an age of 27.1 ± 3.2 years, a height of 1.82 ± 0.1 m, a weight of 71.3 ± 10.4 kg, and a BMI of 21.5 ± 2.6 kg/m^2^. An ophthalmologist examined the participants prior to the measurements.

This study was designed to measure the temporal variations and dependencies of arterial and venous retinal vessel diameters and arterial BP in young and healthy subjects. Inclusion and exclusion criteria were specified to reduce the influence of illness, drugs, stress, and an unhealthy lifestyle. Eyes with cataracts, myopia <-6 dpt, hyperopia >+5 dpt, astigmatism >2 dpt, visual acuity <0.5, or visible changes in fundus were excluded to prevent the effects on the image quality for scaling or reflections. Competitive athletes, subjects with diabetes, cardiovascular disease and hypertension, with an Intraocular pressure (IOP) <9 mmHg, an IOP >21 mmHg, or a BMI below 18.5 or over 30, and smokers were excluded owing to the possible effects on BP and microcirculation. In addition, the consumption of drugs, alcohol, or caffeine was forbidden before the examination, and subjects with an acute infection or a sleep deficit were excluded. Pregnant or breast-feeding women were excluded owing to the unknown impact of Tropicamid on unborn and newborn children. Subjects with glaucoma were excluded owing to the increased risk of a glaucoma attack, and subjects with a known Tropicamid allergy were excluded owing to the increased risk from drug administration. Epileptic subjects were excluded owing to the risk of triggering epileptic shock from a RVA investigation.

After receiving an explanation on the purpose and the details of the study, the subjects gave their written informed consent prior to participation in the study. The data and information from the subjects were anonymized prior to the analysis. The experiments were conducted in accordance with the Declaration of Helsinki. Approval for the study was obtained from the ethics committee of the Friedrich Schiller University of Jena.

### Measurement Setup

**FIGURE 1:**
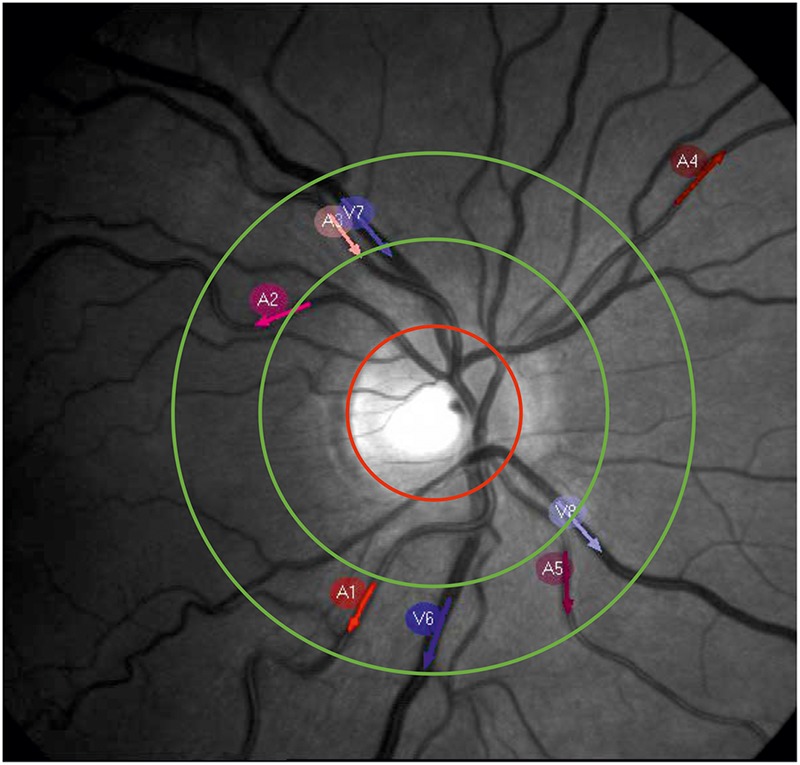
Example picture of a retinal fundus. The red circle marks the optic nerve head. The measurement area for the SVA is located between the green circles. The red and blue arrows show the selected measurement points on the arteries (A1–A5) and veins (V6–V8) for dynamic vessel diameter measurement.

The arterial and venous retinal vessel diameters were recorded using a RVA (RVAResearch, IMEDOS Systems GmbH, Jena, Germany). The measurements were performed on the subjects sitting in front of the fundus camera (Zeiss FF450plus, Carl Zeiss Meditec AG, Jena, Germany) in the eye with greater visual acuity or lower defective vision. The optic disk was placed in the center of the video, as shown in **Figure 1**. The arteries and veins accepted by the RVA software program were selected for the diameter measurement. The number varied between 3 and 10 vessels (mean of 6.8 ± 2.3), depending on the individual vessel path and image quality. The vessel segments were manually selected as the measurement points. The measurement points were preferred to be in the region defined for the SVA examinations between ½ and 1 disk diameter from optic disk. Neighboring vessel segments were chosen if a measurement within this range was not possible. The vessel segments were automatically followed, and their diameters were automatically measured in a video stream by the system software program. The vessel diameters were measured in measuring units, an arbitrary unit that corresponds to micrometers measured in a normal eye. A baseline of the vessel diameters without stimulation and without triggered breathing was recorded for 360 s with 25 samples per second (SPS).

Simultaneously, the arterial BP was recorded by a continuous BP monitor (Finometer PRO, Finapres Medical Systems B.V., Amsterdam, The Netherlands), using a finger cuff on the left middle finger. The BP data were acquired at 480 s with 200 SPS.

The measurement began with BP acquisition. The RVA measurement was started after 60 s. BP acquisition stopped 60 s after the end of the RVA measurement. The measurements were synchronized using a trigger signal produced by the RVA and recorded by a Finometer PRO. The measurement was repeated six times per subject within a time range of 90 min.

### Data Analysis

#### Data Characteristics

The measurement data showed specific characteristics that required special processing methods. The most common and typical artifact types are listed in **Table 1**.

**Table 1:** Typical artifact types in vessel diameter signals (RVA) and BP signal (BP) from Finometer Pro.

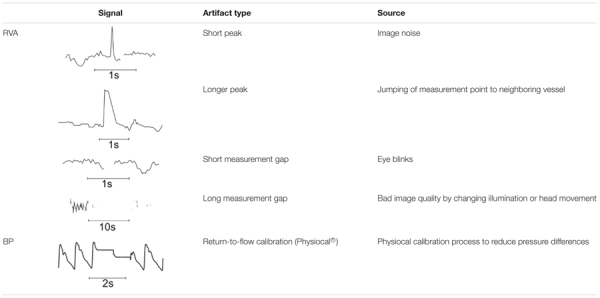

#### Data Selection

The recorded data of the arterial and venous vessel diameters contained several artifacts. The artifacts originated from temporary measurement failures owing to closed eyes, eye movements, low image contrast, or bad illumination of the eye. Some of the artifacts affected a portion of the measurement points or became more frequent toward the end of a measurement because of the declining attention of the subject.

Selection of the data was performed manually. The data from the measurement points were excluded if the amplitude of the HF noise was at least twice the amplitude of the noise in the other data sets. The data were also excluded if the number of long measurement failures was significantly higher than that in the data from other measurement points, or the diameter signal showed rectangular bounds originating from the temporary measurement of an adjacent vessel. The prominent artifacts at the beginning or the end of the dataset were removed by excluding the data segments if the number of artifact samples exceeded the number of valid samples in the signal segment. A minimum of one artery and one vein per measurement was used for the analysis.

**FIGURE 2:**
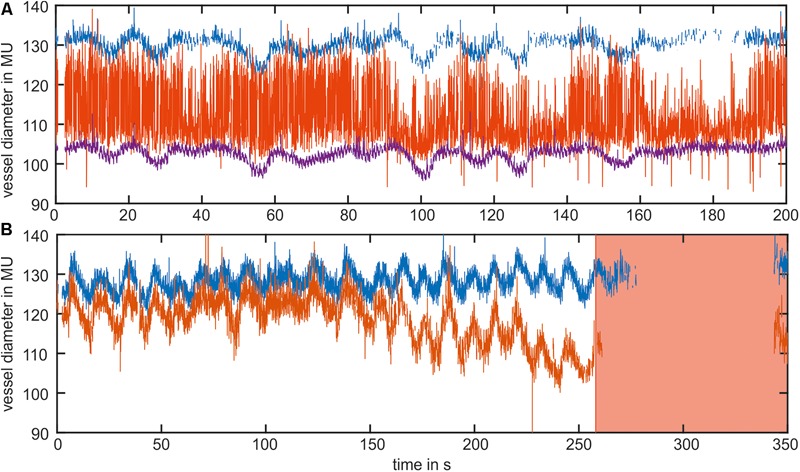
Example of the manual data selection in two datasets of the arterial vessel diameters. **(A)** The blue and purple signals contained an adequate signal quality and were used for the analysis. The red signal contained high noise and was excluded from the analysis. **(B)** Shows the signals with a long measurement gap at the end of the measurement. The red marked area was excluded from the analysis.

An example of the manual data selection for two typical datasets of the arterial vessel diameters is shown in **Figure 2**. **Figure 2A** shows the diameter signals of three arteries. The purple signal demonstrates an adequate signal quality with low noise and a small number of measurement gaps. The blue signal exhibited several short measurement gaps that could be connected by interpolation. Therefore, the blue signal could also be included in the data analysis. In contrast, the red signal contained high noise derived from the measurement errors and was excluded from the analysis. **Figure 2B** shows diameter signals of two arteries containing a large measurement gap toward the end of the measurement. For the large amount of missing data, the gap could not be closed by interpolation, and the last 100 s of the signal were excluded from the analysis.

From the BP data, sections of 420 s were selected to remove the artifacts at the beginning of the measurement. The remaining signal selection began at 30 s before the RVA measurement onset and ended 30 s after the RVA measurement. The amount of the BP data samples was not affected by a potential length reduction of the RVA signal.

#### Pre-processing

The short peaks in the RVA data were removed by median filtering with a filter length of three samples (0.12 s). The gaps in the measurement data were filled using linear interpolation. The peaks exceeding the median filtered signal (filter length: 51 samples, 2.04 s) by more than the SD of the original signal were eliminated by interpolation.

**FIGURE 3:**
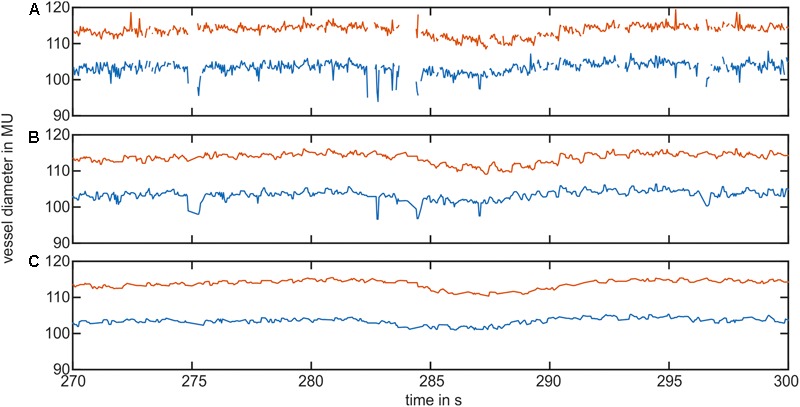
Example of the vessel diameter signal interpolation and artifact correction. **(A)** Shows the original vessel diameter signal containing peaks and measurement gaps. **(B)** Shows the signal after interpolation and the first median filter. **(C)** Shows the vessel diameter signal after removing short signal peaks.

**Figure 3** shows the pre-processing of the selected vessel diameter data. The raw data in **Figure 3A** contained measurement gaps and short peaks in the vessel diameter signal. After interpolation and first median filtering, the signal in **Figure 3B** was free of gaps or short peaks; however, the signal contained longer peaks. **Figure 3C** shows the signal after using the peak removal algorithm. The longer lasting nonphysiological peaks were removed.

The measurement artifacts were removed, and the oscillations caused by pulsation were reduced in the vessel diameter signal. However, the slower variations in the VLF and LF ranges were contained in the measurement signal. After artifact reduction, the included artery diameter signals were combined to obtain one signal for the arteries, and the included vein diameter signals were combined to obtain one signal for the veins.

The BP data contained measurement artifacts owing to physiological recalibration (Physiocal) during the measurement (Wesseling et al., 1995). These calibration sections were automatically recognized by their plane signal shape. They were automatically replaced up to the neighboring diastolic peaks by the mean value of the artifact-free signal.

**FIGURE 4:**
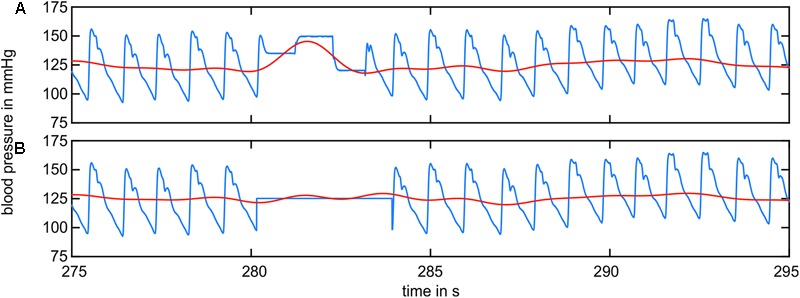
BP signal containing recalibration artifacts (blue) and the filtered BP signal in the LF range (red). **(A)** The raw BP signal contained artifacts from recalibration. The filtered signal shows prominent peaks. **(B)** The recalibration sections were replaced by the mean value of the remaining data. The filtered signal did not show prominent peaks.

The artifact correction in the arterial BP data is shown in **Figure 4**. The original BP signal (blue) shown in **Figure 4A** contained multiple rectangular-shaped signal artifacts owing to recalibration. They occurred once a minute and lasted approximately 2–5 s. The low-pass-filtered signal (LF range, red) showed a massive peak during this artifact. The artifact-affected signal was removed up to the neighboring diastolic peaks and replaced by the mean value of the remaining data (B, blue). The low-pass-filtered signal (red) showed a significant reduction in the artifact-related peak.

#### Frequency Analysis

The maximum amplitudes of vasomotion in the VLF and LF ranges were determined. The VLF range was defined as 0.02–0.07 Hz, and the LF range was defined as 0.07–0.15 Hz based on previous publications (Vermeij et al., 2014; Choi et al., 2015). The pre-processed signal was upsampled to the next power-of-two length (Bach and Meigen, 1999). A fast Fourier transformation was performed. The spectrum was filtered and retransformed using an inverse Fourier transformation. The resulting signal was downsampled to the original time base. The maximum amplitudes were determined as the difference between the maximum and the minimum value of the filtered signal.

#### Time-Frequency Analysis

**FIGURE 5:**
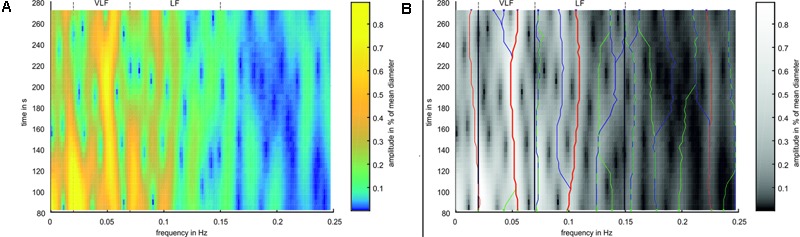
Example for Time-Frequency Analysis. **(A)** Spectrogram of an arterial vessel diameter signal. **(B)** Spectrogram in simplified colors. Green ‘x’ mark the peaks in the first window, blue ‘x’ mark the peaks in the last window. Green lines mark ridges detected in forward time direction, blue lines mark ridges detected in backward time direction, and red lines mark ridges detected in both directions. Thick red lines mark the most prominent ridges in VLF and LF range.

A time-frequency analysis was performed using a spectrogram. The artery and vein diameter signals were windowed using a hamming window with a length of 2^12^ samples (approximately 164 s). The signal was extended for the Fourier transformation to 2^14^ samples by zero padding. The window was shifted in steps of 125 samples (5 s) between the transformations. For analyzing the BP data, a hamming window length of 2^15^ (approximately 164 s) samples, a signal transformation length of 2^17^, and a shift of 1,000 samples (5 s) were applied with respect to the eight-fold higher sampling rate. A spectrogram of the artery diameters is shown in **Figure 5A**.

The analysis process is shown in **Figure 5B**. Frequency peaks were detected in the spectra of the first (green X) and last (blue X) selected windows. A ridge was determined by detecting the closest peak in the respective adjacent spectra (green and blue lines). A ridge was defined as prominent if the same ridge was detected forward and backward over all spectra (red lines). If multiple prominent ridges were detected in the VLF or LF ranges, the ridge with the highest mean amplitude was selected (bold red lines). For the prominent ridges in the VLF and LF ranges, a statistical analysis was conducted by calculating the mean value and SD of the frequencies and amplitudes within single measurements. These values were compared between all the measurements.

#### Signal Correlation

For the detection of time dependencies between the signals, a cross-correlation analysis was performed. Therefore, artery and vein diameter signals were upsampled to the sampling rate of the BP data by linear interpolation. The signals were filtered to match the VLF or LF ranges using zero phase Butterworth high-pass and low-pass filters. The signals were normalized to a mean value of 0 and an SD of 1. The artery and vein diameter signals were zero-padded to match the signal length of the BP data. This allowed a time shift in cross-correlation of 30 s without a change of the effective data samples in the calculation. The cross-correlation values were computed between the BP and artery diameters and between the BP and vein diameters. The correlation amplitudes were normalized with respect to the length of the vessel diameter data. In the time shift range of -30 to +30 s, the absolute minimum and maximum values and their amplitudes were determined.

## Results

### Signal Amplitudes

**FIGURE 6:**
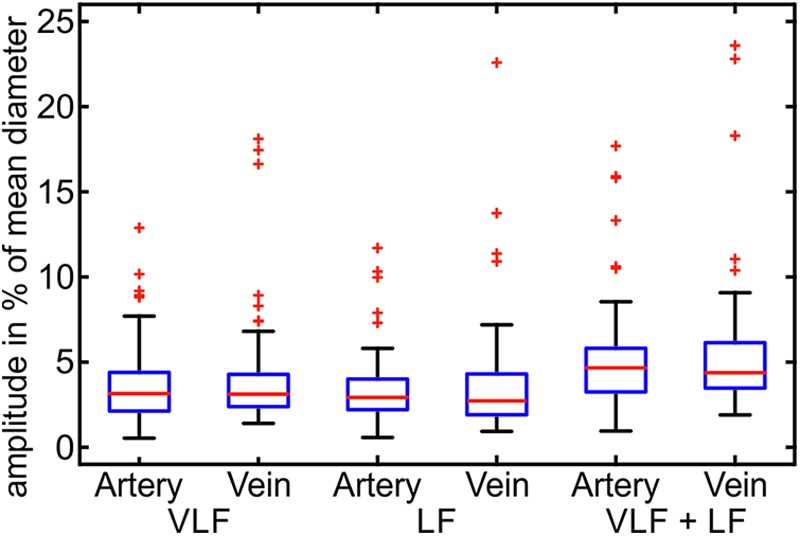
Maximum amplitudes of the Mayer waves in the VLF and LF ranges in the arterial and venous vessel diameters and the arterial BP (*n* = 90).

The artifact-corrected vessel diameter and BP signals were filtered by a fast Fourier transform (FFT) bandpass filter to the determined frequency ranges. The maximum peak-to-peak amplitudes of the signals were determined within the VLF, LF, and VLF + LF ranges. **Figure 6** shows the maximum amplitudes of the artery and vein diameters in relation to their mean vessel diameters.

The maximum variation of the vessel diameter signals was in the range of 0.5–7.7% of the mean diameters for the arteries and veins in the VLF and LF ranges. A few outliers showed significantly higher amplitudes of the vessel diameters. The combined frequency range of the VLF and LF showed slightly higher amplitudes up to 9.5% and a few outliers. The variation between the measurements was high, and the maximum amplitudes varied in a wide range.

The statistical values of the maximum amplitudes are listed in **Table 2**. The mean amplitude was between 3.2 and 4.0% of the mean diameter for the artery and vein diameters in the VLF and LF ranges. When combining the frequency ranges, the amplitudes of the vessel diameters were approximately 5.0–5.7%. The veins showed slightly higher amplitudes in all the frequency ranges. The high SD values showed a high variation between the single measurements. The variations in BP were higher in relation to the mean value than the vessel diameters.

**Table 2:** Statistical analysis of artery and vein diameter and BP amplitudes in the VLF, LF, and VLF + LF ranges.

	Artery (%)	Vein (%)	BP (%)
VLF	3.58 ± 2.19	3.93 ± 2.97	13.59 ± 4.55
LF	3.29 ± 1.95	3.54 ± 2.95	12.97 ± 5.49
VLF + LF	5.08 ± 3.00	5.68 ± 4.53	20.13 ± 6.70

### Time Frequency Analysis

**FIGURE 7:**
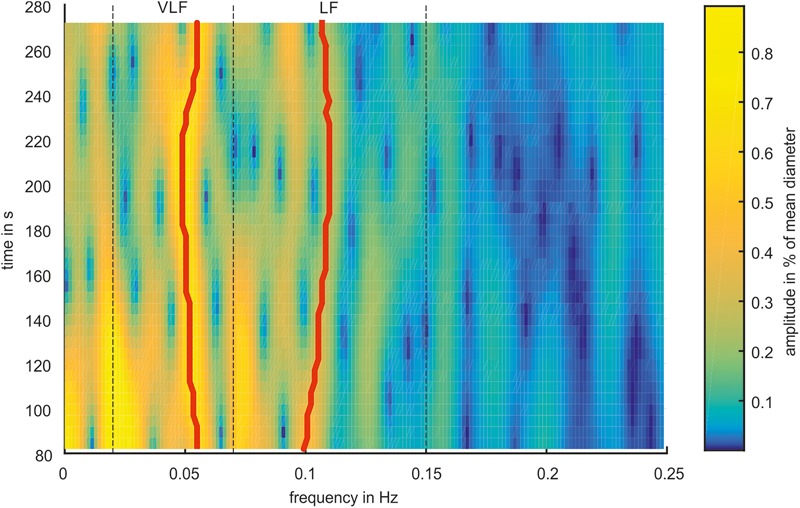
Spectrogram of a representative arterial vessel diameter signal. The prominent frequencies in the VLF and LF ranges are marked by red lines.

The characteristics of the Mayer wave signals were determined using a spectrogram. **Figure 7** shows a spectrogram of a typical arterial vessel diameter signal. The signal contained multiple frequency components. The frequencies and amplitudes of the components varied over time. The frequency of 0.1 Hz and below contained a higher activity than a higher frequency range. The most dominant signals of the VLF and LF ranges are shown with a red line. In this example, the prominent signal in the VLF range contained a frequency between 0.049 and 0.055 Hz and an amplitude between 0.59 and 0.77%. In the LF range, the frequency with the most prominent signal varied between 0.099 and 0.110 Hz, and the amplitude varied between 0.23 and 0.44%. Other considerable activities were visible at approximately 0.2 Hz at the beginning of the measurement; however, the amplitude decreased with increasing time.

**FIGURE 8:**
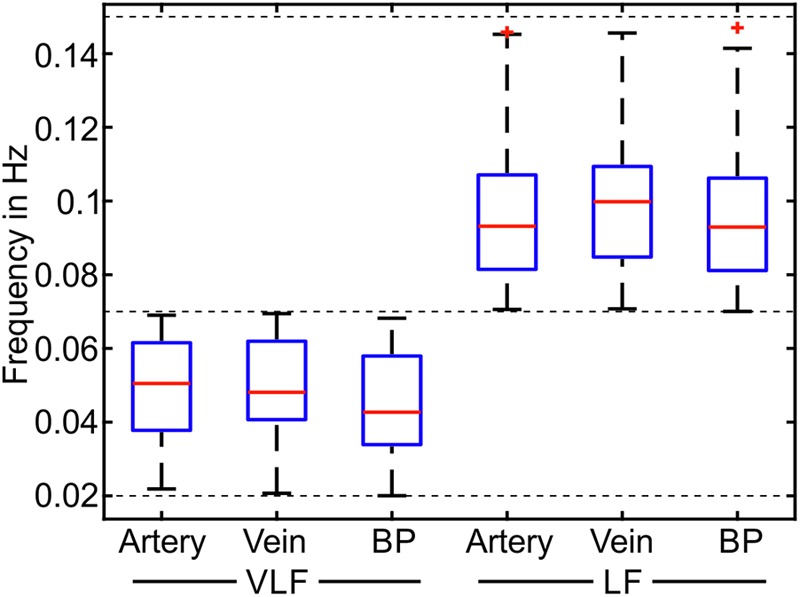
Mean frequencies of the most prominent frequencies for the measurements in the VLF and LF ranges (*n* = 90).

The most prominent signals were selected in the spectrogram of the selected and pre-processed data. The mean frequencies of the prominent signals are shown as boxplots in **Figure 8**. The span of the whiskers showed the broad distribution of the most prominent frequencies. The frequency values were distributed throughout the VLF and LF ranges; however, an accumulation occurred at approximately 0.05 Hz for VLF and 0.1 Hz for LF.

In **Table 3**, the statistical values of the prominent frequencies were compared for the diameters of the arteries and veins, as well as the BP. The VLF and LF means showed the determined mean frequencies within a measurement and their variation between multiple measurements. The VLF and LF SDs showed the SD of the prominent frequencies within single measurements and the variation of the SD between multiple measurements. The statistical analysis showed that the SD of the frequencies between multiple measurements was higher than the SD of the frequencies within single measurements. The values of the prominent frequencies in the arterial BP were in the same range as those in the arterial and venous vessel diameters.

**Table 3:** Statistical evaluation of the frequency values of the most prominent frequencies.

	Artery (Hz)	Vein (Hz)	BP (Hz)
VLF mean	0.0485 ± 0.0139	0.0494 ± 0.0139	0.0443 ± 0.0145
VLF SD	0.0030 ± 0.0016	0.0030 ± 0.0013	0.0034 ± 0.0014
LF mean	0.0978 ± 0.0210	0.1003 ± 0.1924	0.0971 ± 0.0202
LF SD	0.0028 ± 0.0013	0.0029 ± 0.0014	0.0030 ± 0.0014

*For each measurement, the mean frequency and SD within the measurement was determined. The table provides the mean and SD of these values within all the measurements.*

**FIGURE 9:**
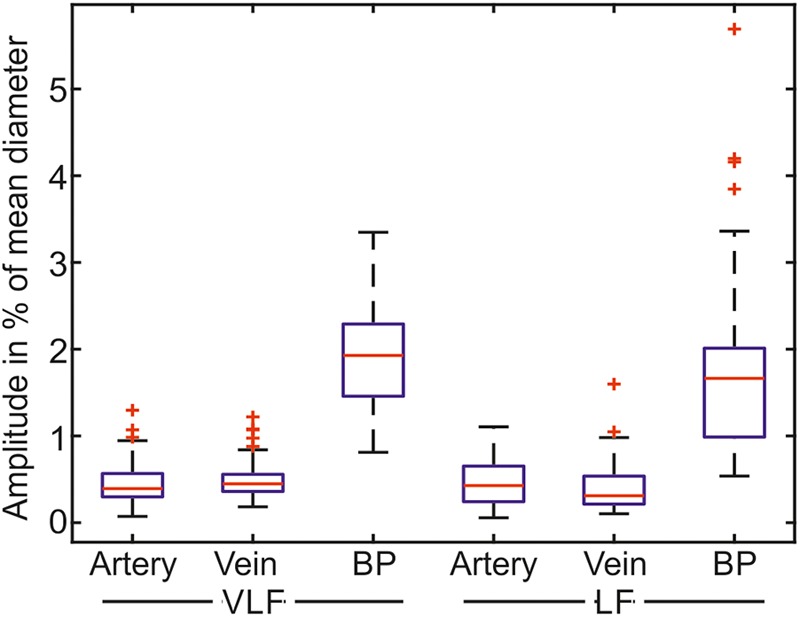
Mean amplitudes of the most prominent frequencies for the measurements in the VLF and LF ranges (*n* = 90).

The mean amplitudes of the prominent frequencies are shown as boxplots in **Figure 9**. The mean amplitude of the most prominent frequencies in the retinal vessels was below 1% of the mean value for most of the retinal vessels. The mean amplitude of the BP was approximately three times the mean amplitude of the vessel diameters.

The statistical values listed in **Table 4** confirmed the low values for the amplitudes of the determined prominent frequencies. The prominent signals in the VLF and LF ranges had approximately equal amplitudes. The SD within the single measurements was high in relation to the mean values. The amplitudes varied within the measurements.

**Table 4:** Statistical evaluation of the amplitudes of the most prominent frequencies.

	Artery (%)	Vein (%)	BP (%)
VLF mean	0.464 ± 0.251	0.496 ± 0.226	1.931 ± 0.625
VLF SD	0.112 ± 0.095	0.117 ± 0.077	0.461 ± 0.255
LF mean	0.442 ± 0.253	0.411 ± 0.268	1.742 ± 0.962
LF SD	0.101 ± 0.066	0.089 ± 0.069	0.403 ± 0.290

*For each measurement, the mean amplitude and SD within the measurement was determined. The table presents the mean and SD of these values within all the measurements.*

### Signal Correlation

**FIGURE 10:**
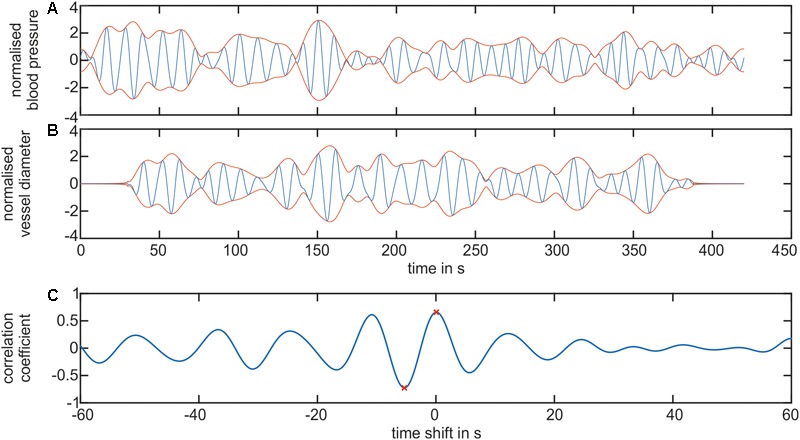
Cross-correlation of the BP and retinal vessel diameters. **(A)** Shows the filtered LF signal of the arterial BP (blue) and the envelope of the signal (red). **(B)** Shows the filtered LF signal of the arterial vessel diameters (blue) and the envelope of the signal (red). **(C)** Shows the cross-correlation signal between the arterial BP and arterial vessel diameter. The global minimum and maximum value is denoted as a red ‘x.’

A cross-correlation analysis was performed to determine the temporal dependencies between the arterial and venous vessel diameters and the arterial BP. **Figure 10** shows the results of a signal processing process of a cross-correlation between the BP and artery diameters in the LF range. **Figure 10A** shows the filtered BP signal. In **Figure 10B**, the filtered arterial vessel diameter signal is shown. The signal extensions from zero padding were visible in the arterial vessel diameter signal. The BP and vessel diameter signals showed a similar signal envelope. The computation of the cross-correlation generated an oscillating signal with a frequency of approximately 0.1 Hz, cf. **Figure 10C**. The most prominent amplitudes were visible at approximately a time shift of 0 s. The global minimum and maximum values of cross-correlation were determined for all the signals. The maximum values depicted the time shift and correlation coefficient for the highest similarity between the BP and vessel diameter signal. However, the minimum values represented the similarity between the BP and the inverted vessel diameter signal. At negative time shifts, BP was leading to vessel diameters and vice versa.

**FIGURE 11:**
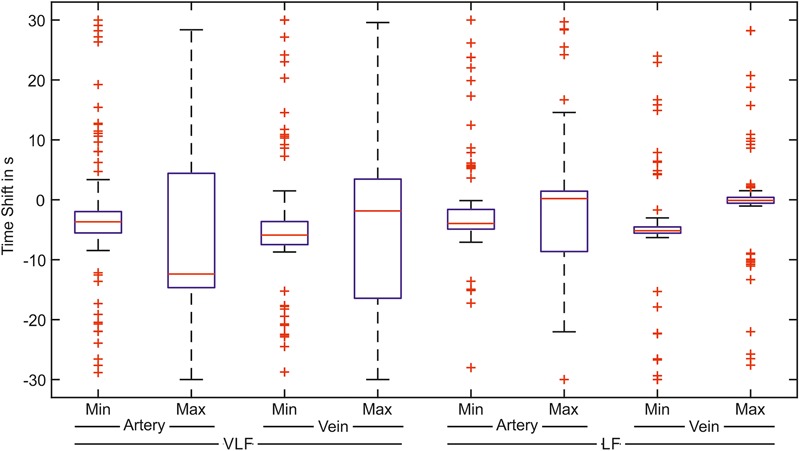
Time shifts of the greatest correlation between the arterial BP and the artery or vein vessel diameters. The box plot shows the minimum and the maximum value of the correlation signal for the correlation with the arteries and veins in the VLF and LF ranges (*n* = 90).

The cross-correlation between the arterial BP and arterial vessel diameters as well as between the arterial BP and venous vessel diameters were investigated in the VLF and LF ranges. The determined time shifts of the minima and maxima of the cross-correlation signals of all the measurements are shown as box plots in **Figure 11**.

In the VLF range, the boxes and whiskers of the maximum correlation between the BP and arteries as well as the veins reached -30 to 30 s. This showed a high variation of the time shift with the best signal correlation. The boxes of the minimum values were considerably smaller than those of the maximum values. However, many outliers were distributed over the time range. In the LF range, the variation of the time shift values was much smaller than those in the VLF range. For the arteries, the minimum values of the cross-correlation were located in a small time shift range. For the cross-correlation between the BP and vein diameters in the LF range, the determined minimum and maximum values were located within a small time shift range. A smaller variation was observed in the maximum values.

**FIGURE 12:**
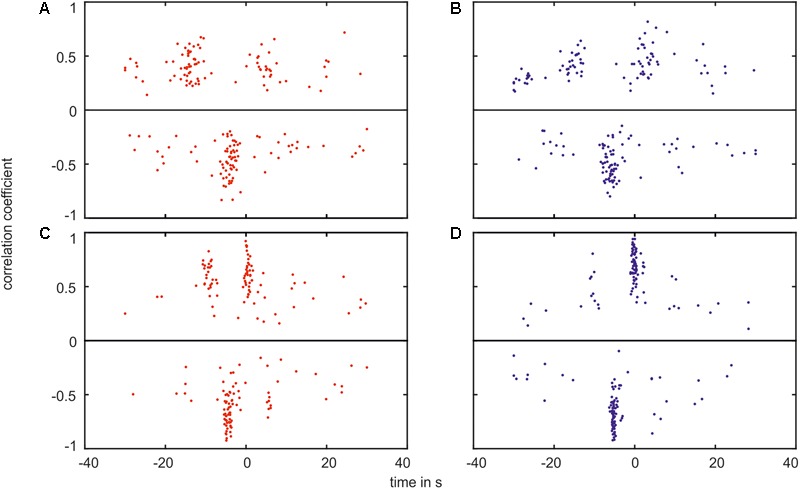
Plot of the correlation coefficient verses the time shift of the greatest correlation. The plot shows the maximum values (positive values, on top of the black line) as well as the minimum values (negative values, below the black line) for the correlation between **(A)** the arterial BP and the arterial vessel diameters in the VLF range, **(B)** the arterial BP and the venous vessel diameters in the VLF range, **(C)** the arterial BP and the arterial vessel diameters in the LF range, and **(D)** the arterial BP and the venous vessel diameters in the LF range.

The box plots show the variation of the values. The distribution of the time shift in relation to the amplitude of the cross-correlation is shown in **Figure 12** for every measurement. Each plot contained the minimum (negative) and maximum (positive) values of cross-correlation.

In the plots, the majority of the data points were located within agglomerations. In the VLF range, shown in **Figures 12A,B**, the agglomerations were approximately -15 and +5 s for the positive values and approximately -5 s for the negative values. The agglomerations contained a distance of approximately 20 s in the time shift. The agglomerations of the negative values were located between the positive values. With an increasing correlation coefficient value, the time shift range decreased. A few data points were randomly distributed and did not belong to an agglomeration. The amplitude of the cross-correlation for these data points was approximately 0. The measured BP and vessel diameter signals did not show a clear correlation.

In the LF range, shown in **Figures 12C,D**, the data points were located in agglomerations with a distance of an approximately 10 s time shift. The variation of the time shift within one agglomeration was smaller than in the VLF range. The positive data points were located at approximately -10 and 0 s. However, the majority of the negative data points were located at approximately -5 s. The connection between the correlation coefficient and the time shift range was more pronounced than in the VLF range.

## Discussion

This study was the first investigation of the temporal dependency between Mayer waves in retinal vessel diameters and arterial BP. The results of the cross-correlation analysis between arterial BP and arterial and venous vessel diameter signals showed temporal dependencies. The minimum spread was seen in the minima for the arteries and veins in the VLF range and in the minima of the arteries and maxima for the veins in the LF range. A minimum in the correlation implies that the arterial BP was similar to the inverted signal of the retinal vessel diameter. A negative time shift implies that the arterial BP was in advance of the retinal vessel diameter. A determined time shift of -5 s for the minimum of the arteries in the LF range indicates that an increase in BP results in a decrease in arterial vessel diameters with a time shift of 5 s. A vascular constriction was caused by a myogenic response to the increase in BP, a quick blood flow autoregulation mechanism. In renal blood flow autoregulation in rats, the myogenic response was shown in a range of 0.1–0.3 Hz and contained a half-life of 1–4 s (Flemming et al., 2001).

The relationship between the determined time shift and correlation power was shown in the LF range for the minimum of the arteries as well as for the maximum of the veins. The measurements with a high correlation between BP and vessel diameters were within a small time shift range. The measurements classified as outliers in boxplot showed a lower correlation. In some diagrams, a cluster of outliers was shifted by 20 s in the VLF range and by 10 s in the LF range. Due to the periodicity of the correlation signal, an adequate correlation at the medium time shift was assumed. A few measurements showed randomized outliers, which could be caused by prominent artifacts.

The retinal vessel diameters were affected by multiple variations. Vasomotion caused by Mayer waves represented a substantial portion of these variations in the arteries and veins in the VLF and LF ranges. This study showed mean variations of 5.08% for the arteries and 5.68% for the veins in the VLF and LF ranges. These values exceeded the variations of 3.71% for the arteries and 2.61% for the veins, determined in a study by Chen (1994). This can be caused by two factors. This study observed all the variations using a dynamic recording over several minutes, whereas Chen et al. determined vasomotion as the spread of the diameter values between three photographs. In addition, the subjects in this study were younger. Thus, a higher influence of vasomotion could be expected (Jarisch et al., 1987). The amplitudes of vasomotion showed large variations between the measurements. Excluding the outliers, the amplitudes reached up to 8.5% for the arteries and 9.0% for the veins. Therefore, the individual variations could be higher than the determined mean values.

A spectrogram analysis determined the most prominent frequencies in the Mayer waves. The determined mean frequencies in the arteries, veins, and BP were similar. The similar frequency values confirmed that identical Mayer waves can be seen in different modalities. The SD of the frequencies between the multiple measurements and multiple subjects was higher than the SD of the prominent frequency within single measurements. Therefore, the individually determined Mayer wave frequencies were more precise and provided more information than the general information for Mayer wave frequencies. The frequencies determined in the arterial BP of 0.0443 ± 0.0145 Hz in the VLF range and 0.0971 ± 0.0202 Hz in the LF range were close to the values of 0.044 ± 0.009 Hz in the VLF range and 0.097 ± 0.021 Hz for the younger adults in a previous study (Vermeij et al., 2014). The amplitudes of the prominent frequencies determined in the spectrogram were smaller than the total signal amplitudes determined in the Section “Time Frequency Analysis.” The spectrogram showed that the vessel diameter and the BP signals consisted of the sum of the frequencies in the LF and VLF ranges. A study investigating Mayer wave oscillations in HRV described subjects with peaky and broadband heart rate spectra (Parati et al., 1995). In this study, most subjects showed broadband spectra without dominating frequency peaks. The total power of vasomotion was distributed over the frequency ranges.

The variation of the frequencies within the single measurements and between the multiple measurements was too high to determine general Mayer wave frequencies. A similarity in the Mayer wave oscillations in the arterial BP and retinal vessel diameters was observed, and the temporal dependency could be determined. Therefore, increasing the reproducibility of the SVA by triggering image acquisition on arterial BP could be investigated. In combination with ECG triggering (Dumskyj et al., 1996), the reproducibility of the SVA could be further increased. An increased repeatability of SVA through additional measurements of arterial BP and ECG could increase the precision of the medical diagnosis using the vessel diameters and AVR. This would lead to a greater acceptance by doctors and a wider distribution in daily clinical practice. Further uses could be in the pulse wave analysis which can be used for the determination of vessel stiffness and diagnosis of hypertension.

## Conclusion

Mayer waves lead to significant variations in retinal vessel diameters. The oscillations consisted of multiple frequencies and varied over time. Due to their high variation, a reliable general statement regarding their frequencies and amplitudes is not possible. The Mayer waves in the retinal vessel diameters showed the same characteristics as in the arterial BP. A temporal dependency between the VLF and LF oscillations in the arterial BP and retinal vessel diameters was shown. Continuous measurements of arterial BP could provide reliable information regarding Mayer waves in retinal vessel diameters.

## Author Contributions

SR, SK, and DB designed the study, interpreted the data, and revised the manuscript. SR analyzed the data and drafted the manuscript.

## Conflict of Interest Statement

The authors declare that the research was conducted in the absence of any commercial or financial relationships that could be construed as a potential conflict of interest.
